# Efficacy, safety, and immunogenicity of rurioctocog alfa pegol for prophylactic treatment in previously treated patients with severe hemophilia A: a systematic review and meta-analysis of clinical trials

**DOI:** 10.12688/f1000research.73884.2

**Published:** 2021-11-11

**Authors:** Bendix Samarta Witarto, Visuddho Visuddho, Andro Pramana Witarto, Henry Sutanto, Bayu Satria Wiratama, Citrawati Dyah Kencono Wungu

**Affiliations:** 1 Medical Program, Faculty of Medicine, Universitas Airlangga, Surabaya, Indonesia; 2 Department of Physiology and Pharmacology, SUNY Downstate Health Sciences University, Brooklyn, New York, USA; 3 Department of Epidemiology, Biostatistics, and Population Health, Universitas Gadjah Mada, Yogyakarta, Indonesia; 4 Graduate Institute of Injury Prevention and Control, Taipei Medical University, Taipei, Taiwan; 5 Department of Physiology and Medical Biochemistry, Universitas Airlangga, Surabaya, Indonesia; 6 Institute of Tropical Disease, Universitas Airlangga, Surabaya, Indonesia

**Keywords:** drug safety, efficacy, hemophilia A, human and medicine, immunogenicity, prophylaxis, rurioctocog alfa pegol

## Abstract

**Background: **Patients with severe hemophilia often present with painful joint and soft tissue bleeding which may restrict them from their daily activities. The current standard of care still relies on a regular prophylactic factor VIII (FVIII), which has a high daily treatment burden. Recently, rurioctocog alfa pegol, a third-generation recombinant FVIII with a modification in its polyethylene glycol (PEG) component, has been developed. Several trials have studied this synthetic drug as bleeding prophylaxis in severe hemophilia A. This study aims to evaluate the efficacy, safety, and immunogenicity of rurioctocog alfa pegol for previously treated patients with severe hemophilia A.

**Methods**: This study was conducted in conformity with the PRISMA guidelines. Data were retrieved from PubMed, Scopus, Cochrane Library, Wiley Online Library, and CINAHL (via EBSCOhost). Study qualities were assessed using the Methodological Index for Non-Randomized Studies (MINORS) and Modified Jadad scales.

**Results:** Four studies involving 517 previously treated severe hemophilia A patients were included in this study. The pooled mean of total annualized bleeding rate (ABR) and hemostatic efficacy was 2.59 (95% CI = 2.04–3.14) and 92% (95% CI = 85%–97%), respectively. Only 30 (2.3%) non-serious and one (1.4%) serious adverse events were considered related to rurioctocog alfa pegol treatment. At the end of the studies, no development of FVIII inhibitory antibodies was observed. None of the developed binding antibodies to FVIII, PEG-FVIII, or PEG was correlated to the treatment efficacy and safety.

**Conclusions:** Despite the limited availability of direct comparison studies, our analyses indicate that rurioctocog alfa pegol could serve as a safe and effective alternative for bleeding prophylaxis in previously treated hemophilia A patients. Moreover, it appears to have low immunogenicity, which further increases the safety profile of the drug in such clinical conditions.

## Introduction

Hemophilia A is a rare, X-linked recessive, congenital bleeding disorder caused by mutations or defects in the factor VIII (FVIII)-producing genes. ^1^ Those mutations manifest as a congenitally absence or decrease of the FVIII, an important pro-coagulant cofactor in the bleeding hemostasis. ^2^ Hemophilia A occurs more commonly than hemophilia B (in 1 out of 5,000 male live births) and accounts for 80% of overall hemophilia cases. ^2^^,^^3^ Hemophilia A may be further classified into mild, moderate, and severe based on the FVIII levels. ^1^ The severe form of hemophilia A is defined as having FVIII levels <1% of normal, while the mild and moderate forms have higher FVIII levels that are approximately 5–50% and 1–5%, respectively. ^3^ Patients with severe hemophilia often present with internal bleeding, especially in the joints and soft tissues. Joint and soft tissue bleeding, along with painful feelings, may restrict patients from their daily activities due to the restriction on their range of motions. ^3^^,^^4^ If this bleeding continues without being treated adequately, hemophilic patients could suffer from more advanced complications, including hemophilic arthropathy. This is important since hemophilic arthropathy could negatively affect their quality of life due to the severe joint immobility. ^3^

The current management of hemophilia A relies on two options: (1) episodic or on-demand FVIII replacement if the patients present with any bleedings to prevent further bleeding or (2) prophylactic FVIII treatment to maintain the FVIII levels and prevent any future bleedings. ^5^ However, the first option was no longer recommended as primary long-term management due to no alteration found in its natural disease course. ^6^ To date, the standard of care for hemophilia A, especially the severe form, still relies on a regular prophylactic intravenous FVIII replacement therapy. Additionally, more than 30% of patients with hemophilia A may develop ‘inhibitors’ or refer to as neutralizing anti-drug antibodies to the standard prophylactic treatment which has high immunogenicity in inducing its formation. ^3^ Thus, extended half-life and safer prophylactic agents may be beneficial in reducing the daily treatment burden, and at the same time, those agents could maintain better clinical presentations and improve the treatment efficacy. ^7^

Recently, rurioctocog alfa pegol (i.e., BAX 855), a third-generation recombinant FVIII (rFVIII) with a modification in its polyethylene glycol (PEG) component, has been developed. ^8^ This modification prolongs the half-life of rFVIII by 1.4–1.5 folds the original rFVIII, thereby reducing the administration frequency and maintaining better bleeding hemostasis of the hemophilic patients. ^8^^,^^9^ Yet, to the best of our knowledge, there are no pooled studies assessing the efficacy, safety, and immunogenicity of rurioctocog alfa pegol. Therefore, here, we aim to evaluate the efficacy, safety, and immunogenicity of rurioctocog alfa pegol, a newly-developed prophylactic agent, in previously treated patients with severe hemophilia A.

## Methods

### Data search strategy

This systematic review and meta-analysis were conducted in accordance with the Preferred Reporting Items for Systematic Reviews and Meta-Analyses (PRISMA) 2009 guidelines. ^10^ A computerized and systematic data searching of relevant studies was conducted in PubMed, Scopus, Cochrane Library, Wiley Online Library, and CINAHL (via EBSCOhost) from inception to 16 February 2021. Keywords were constructed based on Medical Subject Headings (MeSH) terms and other additional terms listed as follows: (“rurioctocog alfa pegol” OR “bax 855” OR “TAK-660” OR “SHP660” OR “adynovate” OR “adynovi”) AND (“hemophilia A” OR “haemophilia A” OR “factor VIII deficiency” OR “factor 8 deficiency” OR “classic hemophilia” OR “classic haemophilia”). Two reviewers searched the literatures independently. Any disagreements were resolved in a consensus involving a third investigator.

### Eligibility criteria

Studies were included if the following criteria were met: (1) study design of clinical trial; (2) study population consists of previously treated severe hemophilia A patients with or without healthy subjects as control; (3) rurioctocog alfa pegol as a prophylactic treatment intervention; and (4) the reported outcomes related to the efficacy, safety, and immunogenicity of rurioctocog alfa pegol (annualized bleeding rate [ABR], patients with zero-bleeding during treatment, hemostatic efficacy, adverse events [AEs], number of deaths, development of FVIII ‘inhibitors’, and/or binding antibodies). The exclusion criteria were as follows: (1) irrelevant titles and abstracts; (2) review articles, systematic reviews, meta-analyses, case reports, case series, letter to editors, and conference abstracts; (3) non-English studies; or (4) irretrievable full-text articles.

### Data extraction and quality assessment

The following relevant data were extracted from the included studies: (1) author and year of publication; (2) study location; (3) clinical trial number; (4) study design; (5) total patients included for prophylactic treatment, gender, and age; (6) definition of target joint (TJ); (7) regimen type; (8) patient characteristics (with or without target joints); (9) total patients in per-protocol analysis set (PPAS) or analyzed for ABR based on regimen type and target joints; (10) outcomes related to efficacy (types of ABR, number of patients with zero-bleeding during treatment, and/or hemostatic efficacy); (11) outcomes related to safety (number of patients with AEs, total AEs, AEs considered related to treatment, and/or number of deaths); and/or (12) outcomes related to immunogenicity (development of FVIII ‘inhibitors’ and/or binding antibodies). The quality assessment of the included studies was performed using the Methodological Index for Non-Randomized Studies (MINORS) scale ^11^ for non-randomized studies and Modified Jadad scale ^12^ for randomized studies. Studies with a MINORS score of ≥ 12 or a Jadad score of ≥ 4 were considered high-quality studies, and the rest were considered low-quality studies. The data extraction and quality assessment were conducted by three reviewers collaboratively through a group discussion and a final decision was taken based on the agreement of all reviewers.

### Statistical analysis

Statistical analyses were performed using the latest version of OpenMeta [Analyst] from the Brown University Evidence-Based Practice Center ^13^ and MetaXL ver. 5.3 (EpiGear International, Sunrise Beach, Australia). Single-arm meta-analysis of mean and standard deviation values was performed for four different efficacy outcomes: (1) total ABR; (2) spontaneous ABR; (3) injury ABR; and (4) joint ABR. Whilst, a meta-analysis of proportions was performed for two different efficacy outcomes: (1) zero-bleeding prevalence and (2) hemostatic efficacy with the rating of excellent or good. Subgroup analysis based on target joints (TJs) for total ABR was also performed. For the purpose of meta-analyses, 95% confidence intervals were transformed into standard deviation values based on a method suggested by the Cochrane Handbook Chapter 6. ^14^

Heterogeneity between studies was assessed with a chi-square test (Cochran’s *Q* statistic) and quantified with the Higgins’ *I* ^2^ statistic. *P*-value < 0.1 from the chi-square test indicated statistical heterogeneity, whereas the level of heterogeneity was determined using *I* ^2^ values. *I* ^2^ < 25% was considered a low heterogeneity, 25–75% a moderate heterogeneity, and *I* ^2^ > 75% a high heterogeneity. If the *I* ^2^ value was greater than 50%, a random-effects model was used for the meta-analysis. Otherwise, a fixed-effects model was applied. Publication bias was explored qualitatively using a funnel plot if the number of studies was adequate (n ≥ 10).

## Results

### Overview of literature search

**Figure 1.**
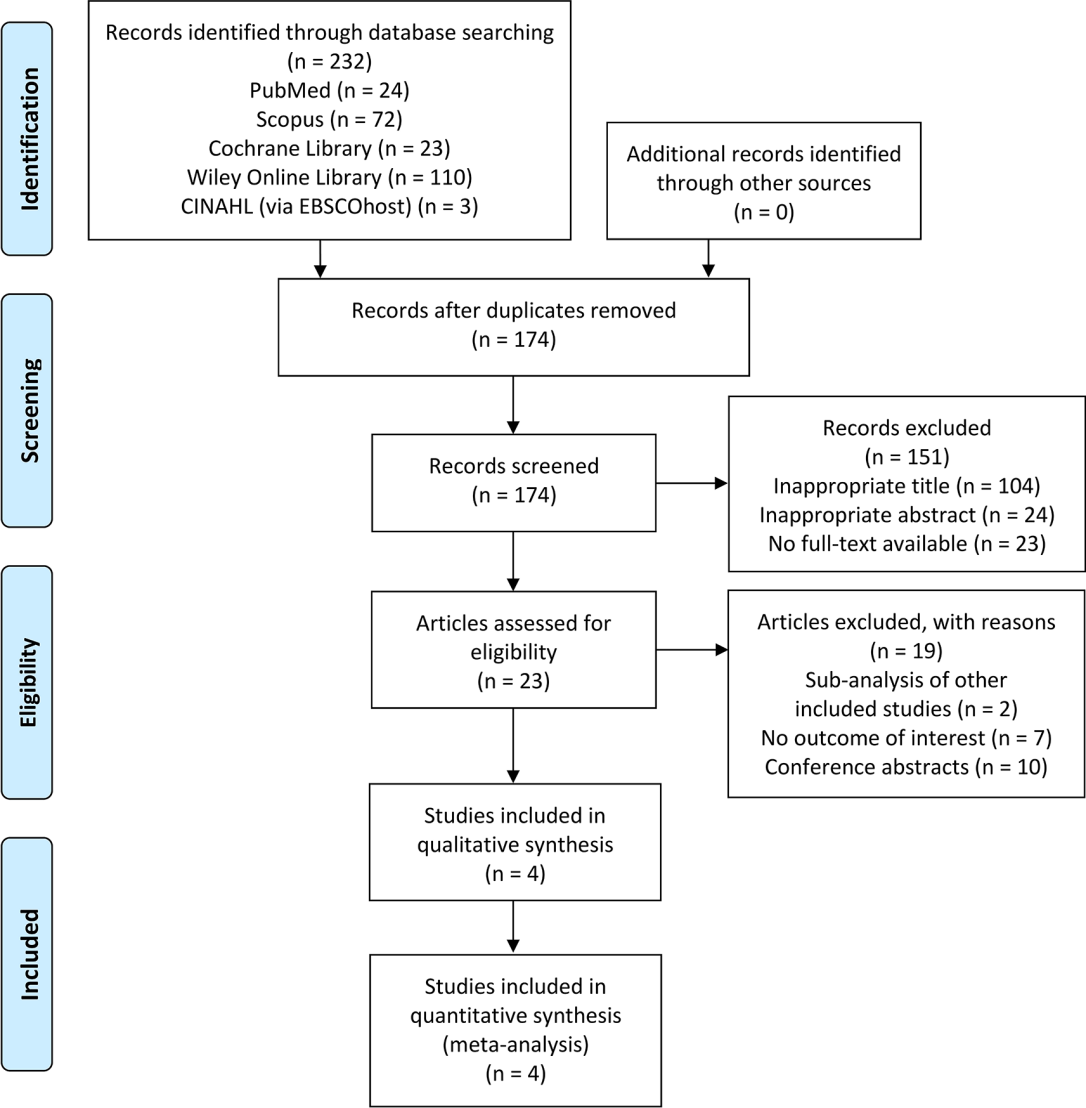
PRISMA flow diagram of the study selection process. PRISMA, Preferred Reporting Items for Systematic Reviews and Meta-Analyses.

The initial search of this study yielded a total of 232 articles identified from PubMed, Scopus, Cochrane Library, Wiley Online Library, and CINAHL (via EBSCOhost). Of those, 174 studies were screened by titles and abstracts after duplicates removal. Twenty-three were fully reviewed based on the eligibility criteria and 19 of these were excluded due to: (1) studies with a sub-analysis of other included studies (n = 2); (2) not reporting the outcome of interest (n = 7); or (3) conference abstracts (n = 10). Finally, four clinical trials ^5^^,^^7^^,^^9^^,^^15^ were included in the qualitative and quantitative synthesis. The overall study selection process is illustrated in Figure 1.

### Characteristics of the included studies

Table 1 provides a summary of the studies included in the systematic review. The four uncontrolled clinical trials ^5^^,^^7^^,^^9^^,^^15^ included a total of 517 previously treated severe hemophilia A patients for prophylactic treatment, with the overall mean ± SD age of 23.9 ± 14.8. Only two studies by Mullins *et al*. ^9^ and Chowdary *et al*. ^7^ included a female patient. The trials were published between 2015 and 2021 and were all multicentered with a range number of 11 to 23 countries. Three ^7^^,^^9^^,^^15^ out of four studies were in phase 3 clinical trial, whereas the study by Konkle *et al*. ^5^ was in a phase 2/3 trial. All studies were non-randomized with the exception of Klamroth *et al*. ^15^ Definition of target joint was the same across all studies, except for Klamroth *et al.* ^15^ There were two different prophylactic regiment types used between studies: twice-weekly and pharmacokinetic (PK)-guided. The “excellent” hemostatic efficacy rating was defined as a complete resolution of pain and sign of bleedings after a single infusion without the requirement of additional infusion to control the bleeding, while the “good” rating was defined when there was a definite improvement in pain and/or signs of bleeding after a single infusion with a possible requirement of more than one infusion to complete the resolution. The “fair” rating was defined as a slight improvement in pain and/or signs of bleeding after a single infusion with definite requirement of more than one infusion to complete the resolution. If there was no improvement or the condition worsen, the hemostatic efficacy was rated “none”. ^5^

**Table 1.** Characteristics of the included studies.

Author, year	Study location	Clinical trial number	Study design	Total patients included for prophylactic treatment (F)	Age *	Definition of target joint
Mullins *et al*., 2017 ^9^	Multicenter (11 countries)	NCT02210091	Phase 3, open-label, non-randomized, uncontrolled clinical trial	66 (1)	6.0 ± 2.7	A joint (ankles, knees, hips or elbows) with ≥ 3 spontaneous bleeding episodes in any consecutive 6-month period
Chowdary *et al*., 2020 ^7^	Multicenter (23 countries)	NCT01945593 (CONTINUATION study)	Phase 3b, open-label, non-randomized, uncontrolled clinical trial	216 (1)	22.8 ± 15.7	A joint with ≥ 3 spontaneous bleeding episodes in any consecutive 6-month period
Konkle *et al*., 2015 ^5^	Multicenter (20 countries)	NCT01736475 (PROLONG-ATE study)	Phase 2/3, open-label, non-randomized, uncontrolled clinical trial	120 (0)	28.7 ± 9.0	A joint with ≥ 3 spontaneous bleeding episodes in any consecutive 6-month period
Klamroth *et al*., 2020 ^15^	Multicenter (22 countries)	NCT02585960 (PROPEL study)	Phase 3, open-label, randomized, uncontrolled clinical trial	57 (0)	31.0 ± 13.6	A joint with ≥ 4 spontaneous bleeding episodes in any consecutive 6-month period
				58 (0)	31.6 ± 12.9	

Author, year	Regimen type	Patient characteristics	Total patients in PPAS or analyzed for ABR based on regimen Type & TJ	Total ABR		Spontaneous ABR		Injury ABR		Joint ABR	
				Mean (95% CI)	SD	Mean (95% CI)	SD	Mean (95% CI)	SD	Mean (95% CI)	SD
Mullins *et al*., 2017 ^9^	Twice-weekly prophylaxis	With TJs	14	3.54 (1.89–6.64)	4.11	1.20 (0.92–1.56)	2.22	2.09 (1.49–2.93)	2.93	1.10 (0.64–1.91)	2.58
		Without TJs	52	2.92 (2.02–4.24)	3.99						
Chowdary *et al*., 2020 ^7^	Twice-weekly prophylaxis	With and without TJs	186	2.23 (1.85–2.69)	3.06	1.20 (0.92–1.56)	2.33	N/A	N/A	1.23 (0.96–1.58)	2.26
	PK-guided prophylaxis	With and without TJs	25	2.64 (1.70–4.08)	1.87	0.96 (0.54–1.71)	0.92			1.40 (0.91–2.17)	0.99
Konkle *et al*., 2015 ^5^	Twice-weekly prophylaxis	With TJs	32	3	4.9	2.2	3.7	N/A	N/A	2.2	3.2
		Without TJs	69	3.7	4.4	1.9	2.9			1.2	2.4
Klamroth *et al*., 2020 ^15^	PK-guided prophylaxis (1–3%)	With and without TJs	52	2.8	3	1.7	2.5	1.1	1.9	1.8	2.2
	PK-guided prophylaxis (8–12%)	With and without TJs	43	1.2	2.4	0.6	1.5	0.7	1.7	0.8	2.3

Author, year	Patients with zero-bleeding during treatment	Hemostatic efficacy			Adverse events						Number of deaths
		Rating	Events	Total number of bleedings	Number of patients with any AEs (non-SAEs and SAEs)	Total non-SAEs	Non-SAEs considered related to treatment	Number of patients with SAEs	Total SAEs	SAEs considered related to treatment	
Mullins *et al*., 2017 ^9^	25	Excellent Good Fair None Not reported	34 29 4 0 3	70	43	152	0	3	4	0	0
Chowdary *et al*., 2020 ^7^	51	Excellent Good Fair None Not reported	438 368 48 4 52	910	174	786	20	33	52	0	1 (considered unrelated to treatment)
Konkle *et al*., 2015 ^5^	40	Excellent/Good Fair/ None/Not reported	498 20	518	73	166	7	5	5	0	0
Klamroth *et al*., 2020 ^15^	24	N/A	N/A	N/A	34	97	2	3	4	0	0
	36				36	98	1	4	5	1	0

Author, year	Development of FVIII inhibitory antibodies	Development of binding antibodies to FVIII / PEG-FVIII/PEG during study
Mullins *et al*., 2017 ^9^	No subjects developed inhibitory antibodies	• 16 developed binding antibodies to FVIII, PEG-FVIII, or PEG prior to exposure, but turned negative while on treatment • 5 developed antibodies to PEG-VIII during treatment (2 were transient; 2 were only at study completion; and 1 was with decreasing titre) • No development of persistent binding antibodies that affected efficacy or safety
Chowdary *et al*., 2020 ^7^	No subjects developed inhibitory antibodies	• 5 developed binding antibodies to FVIII • 8 developed binding antibodies to PEG-FVIII • Only one persisted to the study end without any notable safety or efficacy findings
Konkle *et al*., 2015 ^5^	No subjects developed inhibitory antibodies	• 7 developed transient binding antibodies to PEG-FVIII or FVIII • No subjects developed persistent binding antibodies to FVIII, PEG-FVIII, or PEG • Binding antibodies that were detected could not be correlated to impaired treatment efficacy or related AEs
Klamroth *et al*., 2020 ^15^	No subjects developed inhibitory antibodies	• 3 had single positive binding antibodies to PEG-FVIII and PEG at baseline only • Binding antibodies that were detected could not be correlated to impaired treatment efficacy or related AEs
	1 subject (resolved at the study end)	• 8 developed transient binding antibodies to PEG-FVIII or FVIII. • Binding antibodies that were detected could not be correlated to impaired treatment efficacy or related AEs

* Data are presented in mean ± SD.

ABR, annualized bleeding rate; CI, confidence interval; F, female; FVIII, factor VIII; N/A, not available or not applicable; Non-SAEs, non-serious adverse events; PEG, pegylated; PK, pharmacokinetic; PPAS, per-protocol analysis set; SAEs, serious adverse events; SD, standard deviation; TJ(s), target joint(s).

### Efficacy outcomes

*Total ABR*

**Figure 2.**
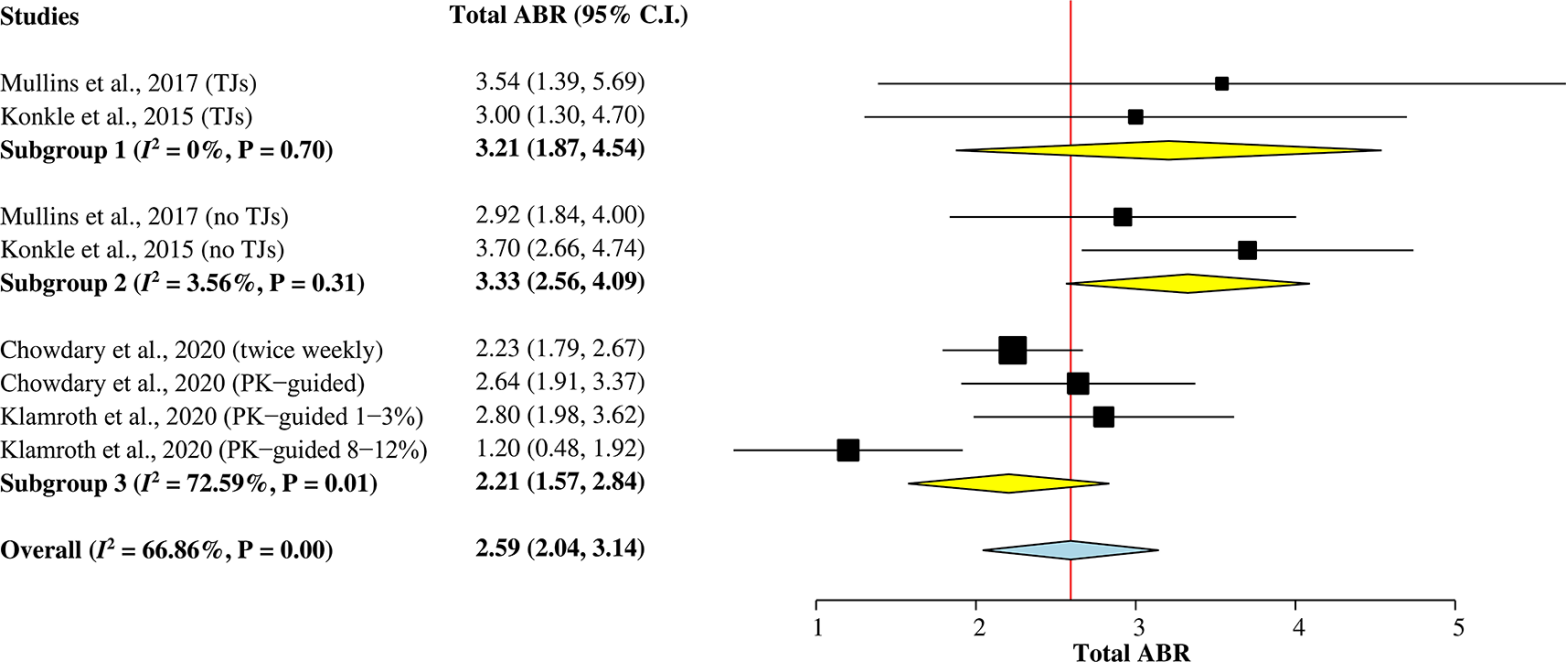
Forest plot of subgroup single-arm meta-analysis for mean of total ABR. ABR, annualized bleeding rate; CI, confidence interval; PK, pharmacokinetic; TJ, target joint.

A total of 473 hemophilia A patients from the four studies ^5^^,^^7^^,^^9^^,^^15^ were included in this subgroup single-arm meta-analysis ( Figure 2) to calculate the pooled mean of total ABR after rurioctocog alfa pegol treatment. A random-effects model was used for the analysis since heterogeneity among studies was greater than 50% ( *I* ^2^ = 67%). The overall pooled mean of total ABR was 2.59 (95% CI = 2.04–3.14).

Two studies ^5^^,^^9^ reporting mean of total ABR individually for patients with target joints (TJs) and without target joints were included in Subgroup 1 and Subgroup 2, respectively. The pooled mean of total ABR in patients with TJs was 3.21 (95% CI = 1.87–4.54), whilst the pooled mean of total ABR in patients without TJs was 3.33 (95% CI = 2.56–4.09). Subgroup 3 included other two studies ^7^^,^^15^ with a combined mean of total ABR for patients with and without TJs. The pooled value was 2.21 (95% CI = 1.57–2.84).

*Spontaneous ABR*

**Figure 3.**
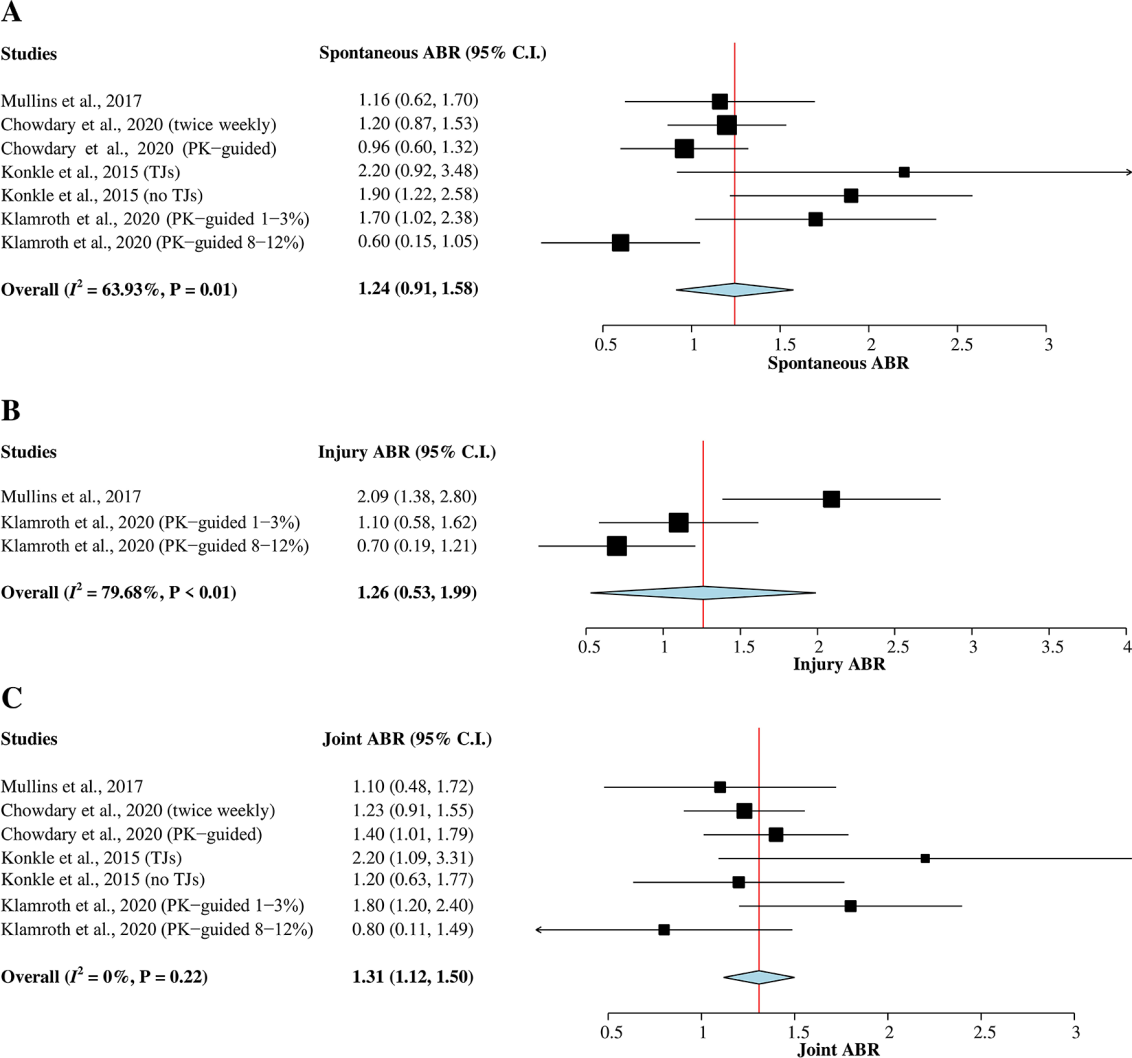
Forest plots of single-arm meta-analysis for (A) mean of spontaneous ABR, (B) mean of injury ABR, and (C) mean of joint ABR. ABR, annualized bleeding rate; CI, confidence interval; PK, pharmacokinetic; TJ, target joint.

The four studies ^5^^,^^7^^,^^9^^,^^15^ with a total of 473 hemophilia A patients were included in this meta-analysis ( Figure 3A). Heterogeneity between studies was greater than 50% ( *I* ^2^ = 64%); therefore, a random-effects model was used for the analysis. The result of the pooled mean of spontaneous ABR was 1.24 (95% CI = 0.91–1.58).

*Injury ABR*

A total of 161 hemophilia A patients from two studies ^9^^,^^15^ that reported mean of injury ABR were included in this meta-analysis ( Figure 3B). A random-effects model was used for the analysis since heterogeneity was greater than 50% ( *I* ^2^ = 80%). The pooled mean of injury ABR was 1.26 (95% CI = 0.53–1.99).

*Joint ABR*

A total of 473 hemophilia A patients from the four studies ^5^^,^^7^^,^^9^^,^^15^ were evaluated in this subgroup analysis of joint ABR ( Figure 3C). The heterogeneity across studies was low ( *I* ^2^ = 0%); therefore, a fixed-effects model was used for the analysis. The pooled mean of joint ABR was 1.31 (95% CI = 1.12–1.50).

*Zero-bleeding prevalence*

**Figure 4.**
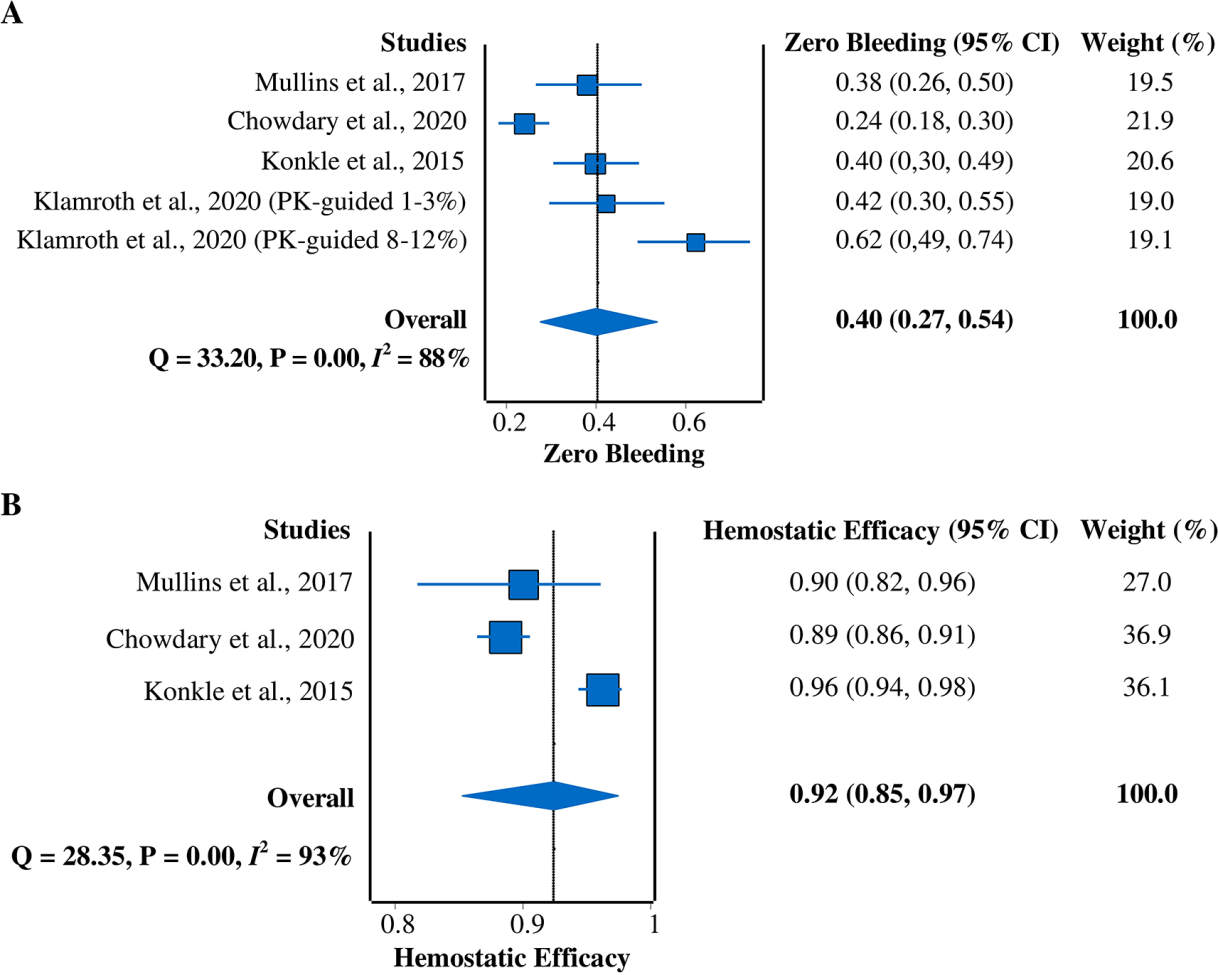
Forest plots of meta-analysis of proportions for (A) zero-bleeding prevalence and (B) hemostatic efficacy (excellent or good rating). CI, confidence interval; PK, pharmacokinetic.

All four studies ^5^^,^^7^^,^^9^^,^^15^ were included in this meta-analysis of zero-bleeding prevalence ( Figure 4A). A random-effects model was used due to the heterogeneity of the data ( *I* ^2^ = 88%). The pooled prevalence result was 40% (95% CI = 27%–54%).

*Hemostatic efficacy*

Three studies ^5^^,^^7^^,^^9^ that reported hemostatic efficacy with the rating of excellent or good were included in this meta-analysis ( Figure 4B). A random-effects model was used due to the heterogeneity across studies ( *I* ^2^ = 93%). The pooled hemostatic efficacy was 92% (95% CI = 85%–97%).

### Safety outcomes

A total of 1,299 non-serious adverse events (non-SAEs) occurred during the four studies. ^5^^,^^7^^,^^9^^,^^15^ However, only 30 (2.3%) of them were considered related to rurioctocog alfa pegol treatment. Whilst, a total of 70 serious adverse events (SAEs) were observed in the four studies and only one (1.4%; a transient development of FVIII inhibitory antibodies) of them – as reported by Klamroth *et al*. ^15^ – were considered related to treatment. Among all studies, only one death case was reported by Chowdary *et al*. ^7^ and was not considered to be related to rurioctocog alfa pegol treatment.

### Immunogenicity outcomes

Three studies ^5^^,^^7^^,^^9^ reported no development of FVIII inhibitory antibodies among all patients. Klamroth *et al*. ^15^ reported one patient with development of FVIII inhibitory antibodies and was resolved at the end of the study. Development of binding antibodies to either FVIII, PEG-FVIII, or PEG among patients was detected in 52 patients from the four studies. However, none of them was correlated to impaired rurioctocog alfa pegol treatment efficacy and AEs.

### Publication bias and quality assessment

Publication bias using funnel plot was not performed due to the low number of the included studies. Details of the quality assessment using MINORS and Modified Jadad scale are provided in Table 2. All non-randomized studies ^5^^,^^7^^,^^9^ were considered high in quality, whereas the randomized study by Klamroth *et al*. ^15^ was considered low in quality.

**Table 2.** Summary of quality assessment using MINORS and Modified Jadad Scale.

MINORS Scale				Modified Jadad Scale	
Items	Mullins *et al*., 2017 ^9^	Chowdary *et al*., 2020 ^7^	Konkle *et al*., 2015 ^5^	Items	Klamroth *et al*., 2020 ^15^
A clearly stated aim	2	2	2	Randomization	1
Inclusion of consecutive patients	2	2	2		
Prospective collection of data	2	2	2	Concealment	0
Endpoints appropriate to the aim of the study	2	2	2		
Unbiased assessment of the study endpoint	0	0	0	Blinding	0
Follow-up period appropriate to the aim of the study	2	2	2		
Loss to follow up less than 5%	2	2	2	Withdrawal or drop-out	1
Prospective calculation of the study size	1	1	1		
**Results**				**Results**	
**Total score**	13	13	13	**Total score**	2
**Study quality**	High	High	High	**Study quality**	Low

MINORS, Methodological Index for Non-Randomized Studies.

## Discussion

This study was the first far-reaching, single-arm meta-analysis that evaluates the efficacy, safety, and immunogenicity of rurioctocog alfa pegol, a newly developed rFVIII product with a prolonged half-life, as a prophylactic treatment for previously treated patients with severe hemophilia A. Rurioctocog alfa pegol (BAX 855) is a pegylated full-length rFVIII product designed to reduce the frequency of prophylactic infusions while maintaining hemostatic efficacy in patients with hemophilia. ^16^^,^^17^ This study indicated the long-term safety and efficacy of the pharmacological agent, which were consistent with the study of rurioctocog alfa pegol for perioperative hemostasis in hemophilia A patients, ^18^^,^^19^ also with the previous parent studies. ^20^^–^^22^

The overall pooled mean of total ABR of rurioctocog alfa pegol is lower compared to the several conventional rFVIIIs ( *Advate* ^®^, *Xyntha* ^®^, *Novoeight* ^®^, *REFACTO* ^®^) with their total ABR ranged from 3.3 to 6.5. ^23^ This could indicate that rurioctocog alfa pegol has advantages over conventional recombinant antihemophilic FVIII. The ABRs were also similar for spontaneous and injury-related bleeding. Any reduction in joint bleeds is considered an improvement in quality of life for hemophilia patients. ^24^ Decreased bleeding in joints thereby shows better joint health, activity, and satisfaction for the patients. ^25^ The mean ABR for patients with target joints was similar to those without target joints, indicating that rurioctocog alfa pegol had an equal efficacy for both groups of patients. Moreover, all studies reported that rurioctocog alfa pegol had higher good and excellent hemostatic efficacy events. This data was comparable with results reported for other rFVIII preparations. ^26^^–^^29^ The efficacy of rurioctocog alfa pegol was also supported by the finding on the pooled zero-bleeding prevalence.

Our study also demonstrated the safety of rurioctocog alfa pegol in patients by assessing the non-SAEs and SAEs. Rurioctocog alfa pegol was also proven to be acceptable and safe for perioperative hemostasis in patients with hemophilia A, with minor findings in both non-SAEs and SAEs. ^18^ Our data showed that most of the adverse reactions were mild. Additionally, rFVIII usage decreased the risk of blood-borne infections and restored longer life expectancies. ^30^ This extended half-life recombinant also improved adherence to prophylactic regimen and reduced the burden of treatment. ^31^^,^^32^ However, there are some concerns regarding the safety of PEG component, particularly when it is used for lifelong prophylaxis. Nevertheless, the PEG levels found in rurioctocogalfa pegol were minimal (less than 1 mg/dose) and evidence has showed no specific side effects to central nervous system, liver, or kidney. ^42^

The development of FVIII ‘inhibitors’ is a major issue in patients treated with blood coagulation factor products. The development of neutralizing alloantibodies against FVIII can reduce the treatment benefits. ^33^^,^^34^ Currently available studies revealed some predictors of ‘inhibitor’ development, but the predictive power remained low. ^35^^,^^36^ Some studies also reported either transient or persistent ‘inhibitor’ development in patients treated with plasma-derived FVIII. ^37^^,^^38^ However, our findings showed no development of persistent FVIII inhibitory antibodies, and this was consistent with the US Food and Drug Administration's approval of rurioctocog alfa pegol for the treatment of hemophilia A patients. ^39^ There was some development of binding antibodies observed. However, this development did not interfere with rurioctocog alfa pegol treatment safety and efficacy until the end of the study.

Overall, our study successfully demonstrated the pooled efficacy, safety, and immunogenicity of rurioctocog alfa pegol as a treatment for hemophilia A. These results can be used to plan an alternative treatment for hemophilia A patients. Nevertheless, high heterogeneity existed between the included studies. We used the random-effects model to minimize this issue. Substantial efforts were made to explore the possible source for heterogeneity, revealing that different dose regimens and prior prophylactic drugs for treatment could be responsible for the high heterogeneity. Regarding the zero-bleeding prevalence ( Figure 4A), a difference in the administered dose regimens was observed among studies. Different dose regimens were considered because pharmacokinetic profiles, targets of FVIII level, and age group varied among patients. The PK-guided dosing was applied to provide more individualized prophylaxis according to each patient’s PK profiles (e.g., plasma half-life), targets of FVIII level, and body weight. Age is also a determining factor since it influences the PK profiles. ^6^

Several other limitations exist in this meta-analysis. First, our study only included single-arm clinical trials. The highest possible quality cannot be ensured due to the lack of control arms. However, since hemophilia is a rare genetic disease, comparison with a control arm receiving prophylaxis with other conventional FVIII products was not recommended, as stated by the regulatory guide. ^40^ Second, diverse prior prophylactic strategies in the patients before switching to rurioctocog alfa pegol may affect the treatment outcomes. Finally, only a few published studies were evaluated in this meta-analysis since rurioctocog alfa pegol is a newly-developed drug. However, these limitations were partly compensated by the multicentered settings of the included studies.

## Conclusions

Our study suggests that rurioctocog alfa pegol is effective, safe, and has low immunogenicity for previously treated patients with severe hemophilia A. Despite the lack of direct comparison studies, rurioctocog alfa pegol could serve as an alternative bleeding prophylaxis in hemophilia A. A network meta-analysis with a multi-arm approach on hemophilia A treatment is warranted to corroborate the current evidence.

## Data availability

All data underlying the results are available as part of the article and no additional source data are required.

## Reporting guidelines

Open Science Framework: PRISMA Checklist for “Efficacy, Safety, and Immunogenicity of Rurioctocog Alfa Pegol for Prophylactic Treatment in Previously Treated Patients with Severe Hemophilia A: A Systematic Review and Meta-Analysis of Clinical Trials”. http://doi.org/10.17605/OSF.IO/7MNRP. ^41^

Data are available under the terms of the Creative Commons Zero “No rights reserved” data waiver (CC0 1.0 Public domain dedication).
