# Supplementary material for: Efficacy, safety, and immunogenicity of rurioctocog alfa pegol for prophylactic treatment in previously treated patients with severe hemophilia A: a systematic review and meta-analysis of clinical trials
Source: F1000Res. 2022 Jan 13;10:1049. Originally published 2021 Oct 15. [Version 3] doi: 10.12688/f1000research.73884.3 (PMC8787562; doi:10.12688/f1000research.73884.3)
Supplement: Peer review history

# Peer review: Reviewer response for version 3

**DOI:** 10.5256/f1000research.119854.r119618

**Published:** 2022-01-24

**Authors:** Claudia Djambas Khayat

**Affiliations:** 1 Hospital Hôtel Dieu de France, Saint Joseph University, Beirut, Lebanon

The modifications done in the manuscript satisfy me. I have no further comments to make.

Are the rationale for, and objectives of, the Systematic Review clearly stated?

Yes

Is the statistical analysis and its interpretation appropriate?

Yes

Are sufficient details of the methods and analysis provided to allow replication by others?

Yes

Are the conclusions drawn adequately supported by the results presented in the review?

Yes

Reviewer Expertise:

Hemophilia and rare Bleeding disorders

I confirm that I have read this submission and believe that I have an appropriate level of expertise to confirm that it is of an acceptable scientific standard.

# Peer review: Reviewer response for version 2

**DOI:** 10.5256/f1000research.79147.r99803

**Published:** 2021-12-03

**Authors:** Claudia Djambas Khayat

**Affiliations:** 1 Hospital Hôtel Dieu de France, Saint Joseph University, Beirut, Lebanon

The authors aim to evaluate the efficacy, safety, and immunogenicity of rurioctocog alfa pegol, in previously treated patients with severe hemophilia A through a systematic review and meta-analysis. Unfortunately, the study only included only 4 single-arm, industry sponsored, clinical trials. This is a significant limitation for the study conclusion. Real life data and non-sponsor studies are needed to set up a strong meta-analysis

 Several remarks concerning the study:

- The introduction is not adequate for the subject. The introduction to hemophilia should be more concise. It will be better to discuss more through the introduction of the importance and burden of regular prophylaxis and the theoretical importance of extended half life in general.
- Concerning the eligible studies. In the discussion you state that this study indicated the long-term safety and efficacy of the pharmacological agent, which were consistent with the study of rurioctocog alfa pegol for perioperative hemostasis in hemophilia A patients [18,19]. - Why were these 2 studies not included in the meta-analysis for safety and efficacy?
- “The overall pooled mean of total ABR of rurioctocog alfa pegol is lower compared to the several conventional rFVIIIs”. - You should have also compared rurioctocog alpha pegol to the other extended half-life product.
- “This extended half-life recombinant also improved adherence to prophylactic regimen and reduced the burden of treatment”. - This statement is not shown in your result.
- You add a new reference in the discussion - a very good one but it will be better to correct the numbering of the reference.
- “The development of FVIII ‘inhibitors’ is a major issue in patients”, “our findings showed no development of persistent FVIII inhibitory antibodies” - You should discuss the fact that all patients are previously treated patients. You are not selling a product, you should do meta-analysis for scientific purposes.
- In fact, the discussion is a little bit superficial. There is no real discussion, just putting the result in a different way.

This article does not have additional value to what is already written like it is.

Are the rationale for, and objectives of, the Systematic Review clearly stated?

Yes

Is the statistical analysis and its interpretation appropriate?

Yes

Are sufficient details of the methods and analysis provided to allow replication by others?

Yes

Are the conclusions drawn adequately supported by the results presented in the review?

Yes

Reviewer Expertise:

Hemophilia and rare Bleeding disorders

I confirm that I have read this submission and believe that I have an appropriate level of expertise to confirm that it is of an acceptable scientific standard, however I have significant reservations, as outlined above.

# Response

**Published:** 2021-12-28

**Authors:** Citrawati Wungu

**Affiliations:** Airlangga University, Indonesia

We would like to thank the reviewer for reading and commenting on our submission. We will attempt to answer each question and suggestion as clearly as possible. Hopefully, revisions made on the manuscript would allow further considerations on indexing the article.

**Reviewer 2**

 Thank you for reviewing our manuscript. We hereby list the responses and revisions made on the original manuscript:

 1. The introduction is not adequate for the subject. The introduction to hemophilia should be more concise. It will be better to discuss more through the introduction of the importance and burden of regular prophylaxis and the theoretical importance of extended half life in general.

**Answer: **Thank you for the suggestion. We have removed unnecessary information to shorten the introduction to hemophilia and added additional information regarding the importance, burden, and theoretical importance of extended half-life rFVIII in the introduction section.

 2. Concerning the eligible studies. In the discussion you state that this study indicated the long-term safety and efficacy of the pharmacological agent, which were consistent with the study of rurioctocog alfa pegol for perioperative hemostasis in hemophilia A patients [18,19]. - Why were these 2 studies not included in the meta-analysis for safety and efficacy?

**Answer: **We did not include the 2 studies since the study aimed to specifically review the role of rurioctocog alfa pegol as a prophylactic treatment. We acknowledged that treatment is different from perioperative hemostasis, in which treatment for hemophilia A considers more regarding the management of patients to prevent their daily bleedings. In order to focus the paper to the prophylactic properties of rurioctocog alfa pegol, we excluded them in this systematic review.

 3. “The overall pooled mean of total ABR of rurioctocog alfa pegol is lower compared to the several conventional rFVIIIs”. - You should have also compared rurioctocog alpha pegol to the other extended half-life product.

**Answer: **Thank you for the insightful feedback. We have added additional comparison of rurioctocog alfa pegol with other extended half-life products in the discussion section.

 4. “This extended half-life recombinant also improved adherence to prophylactic regimen and reduced the burden of treatment”. - This statement is not shown in your result.

**Answer: **Thank you for the constructive comment. We would like to apologize for the unclarity of the sentence. This sentence was not related to the study result and was intended to add information regarding the advantage of extended half-life rFVIIIs products, such as rurioctocog alfa pegol. In regard to this, we would like to revise and correct the structure of the mentioned sentences to avoid misunderstanding.

 5. You add a new reference in the discussion - a very good one but it will be better to correct the numbering of the reference.

**Answer: **Thank you for the meticulous observation. We would like to apologize as there were errors during the numbering input. We have revised the numbering of the references to the correct order.

 6. “The development of FVIII ‘inhibitors’ is a major issue in patients”, “our findings showed no development of persistent FVIII inhibitory antibodies” - You should discuss the fact that all patients are previously treated patients. You are not selling a product, you should do meta-analysis for scientific purposes.

**Answer: **Thank you for the valuable suggestion. We acknowledged that we have not sufficiently discussed regarding the immunogenicity properties of rurioctocog alfa pegol since the patients are all previously treated. We have added additional information in the discussion section. We hope that the revised discussion could provide more objective perspective in choosing treatment for hemophilia A patients.

 7. In fact, the discussion is a little bit superficial. There is no real discussion, just putting the result in a different way. This article does not have additional value to what is already written like it is.

**Answer: **We really appreciate this very useful feedback as well and we have added more contents for the discussion. We hope that the added information are sufficient to provide more in-depth discussion.

**Additional Information**

 We thank the reviewer for giving us an opportunity to improve our manuscript. We have added an additional analysis, the Egger’s test, to explore the potential of publication bias in all outcomes. Accordingly, we included additional details in the method, result, and discussion sections regarding the Egger’s test. We hope that this insertion could further provide additional value to the study findings.

# Peer review: Reviewer response for version 2

**DOI:** 10.5256/f1000research.79147.r99792

**Published:** 2021-11-12

**Authors:** Yelvi Levani

**Affiliations:** 1 Faculty of Medicine, Universitas Muhammadiyah Surabaya, Surabaya, Indonesia

The author has been revised the article according to reviewer's comment. Therefore, no further comments needed.

 Thank you.

Are the rationale for, and objectives of, the Systematic Review clearly stated?

Yes

Is the statistical analysis and its interpretation appropriate?

I cannot comment. A qualified statistician is required.

Are sufficient details of the methods and analysis provided to allow replication by others?

Yes

Are the conclusions drawn adequately supported by the results presented in the review?

Yes

Reviewer Expertise:

Immunology

I confirm that I have read this submission and believe that I have an appropriate level of expertise to confirm that it is of an acceptable scientific standard.

# Peer review: Reviewer response for version 1

**DOI:** 10.5256/f1000research.77573.r97267

**Published:** 2021-11-02

**Authors:** Yelvi Levani

**Affiliations:** 1 Faculty of Medicine, Universitas Muhammadiyah Surabaya, Surabaya, Indonesia

This paper is clearly written and well organized. This paper presented the meta analysis of the efficacy, safety and immunogenicity of newly developed drug for severe hemophilia A, rurioctocog alfa pegol. The figures and tables are also clear and helpful.

 However, there are some parts that need clarifying:

- In the result part (section: safety outcomes) the author stated: *"A total of 1,299 non-serious adverse events (non-SAEs) occurred during the four studies. ^5^^,^^7^^,^^9^^,^^15^ However, only 30 (2.3%) of them were considered related to rurioctocog alfa pegol treatment. Whilst, a total of 70 serious adverse events (SAEs) were observed in the four studies and only one (1.4%) of them – as reported by Klamroth et al. ^15^ – were considered related to treatment" -* I think it would be nicer, if author gives more explanation or examples about what kind of serious adverse that related to rurioctocog alfa pegol treatment.
- In the discussion part, the author stated, *"Regarding the zero-bleeding prevalence (Figure 4A), a difference was observed among studies that employed different dose regimens. Different dose regimens were considered because pharmacokinetic profiles, targets of FVIII level, and age group varied among patients." - *I think it would be better if the authors explain more about the dose regimens and pharmacokinetic in the introduction or discussion part.

I added a reference from Stidl *et aI.* (2018) ^1^, because the article explained about the safety and potential adverse effects that related to rurioctocog alfa pegol. This reference could help enrich the discussion of article, especially in the part of safety and adverse effects from the drug.

 Overall, this article is interesting and gives valuable information.

Are the rationale for, and objectives of, the Systematic Review clearly stated?

Yes

Is the statistical analysis and its interpretation appropriate?

I cannot comment. A qualified statistician is required.

Are sufficient details of the methods and analysis provided to allow replication by others?

Yes

Are the conclusions drawn adequately supported by the results presented in the review?

Yes

Reviewer Expertise:

Immunology

I confirm that I have read this submission and believe that I have an appropriate level of expertise to confirm that it is of an acceptable scientific standard.

## References

[1] : Polyethylene Glycol Exposure with Antihemophilic Factor (Recombinant), PEGylated (rurioctocog alfa pegol) and Other Therapies Indicated for the Pediatric Population: History and Safety. *Pharmaceuticals (Basel)* .2018;11(3) : 10.3390/ph11030075 10.3390/ph11030075 30049994PMC6160981

# Response

**Published:** 2021-11-04

**Authors:** Citrawati Wungu

**Affiliations:** Airlangga University, Indonesia

We would like to thank the reviewer for reading and commenting on our submission. We will attempt to answer each question and suggestion as clearly as possible. Hopefully, revisions made on the manuscript would allow further considerations on indexing the article.

 We hereby list the responses and revisions made on the original manuscript:

 1. In the result part (section: safety outcomes) the author stated: "A total of 1,299 non-serious adverse events (non-SAEs) occurred during the four studies. ^5,7,9,15^ However, only 30 (2.3%) of them were considered related to rurioctocog alfa pegol treatment. Whilst, a total of 70 serious adverse events (SAEs) were observed in the four studies and only one (1.4%) of them – as reported by Klamroth *et al*. ^15^ – were considered related to treatment" - I think it would be nicer, if author gives more explanation or examples about what kind of serious adverse that related to rurioctocog alfa pegol treatment.

**Answer: **Thank you for the suggestion. We have added an additional information regarding the serious adverse event occurred related to the treatment as reported by Klamroth *et al.* ^15^ in the safety outcomes section of the result part.

 2. In the discussion part, the author stated, "Regarding the zero-bleeding prevalence (Figure 4A), a difference was observed among studies that employed different dose regimens. Different dose regimens were considered because pharmacokinetic profiles, targets of FVIII level, and age group varied among patients." - I think it would be better if the authors explain more about the dose regimens and pharmacokinetic in the introduction or discussion part.

**Answer: **We have provided further explanation regarding the dose regimens and pharmacokinetic in the discussion part. We would also like to revise and correct the structure of the mentioned sentences to avoid unclarity.

 3. I added a reference from Stidl et aI. (2018) ^1^, because the article explained about the safety and potential adverse effects that related to rurioctocog alfa pegol. This reference could help enrich the discussion of article, especially in the part of safety and adverse effects from the drug.

 Stidl R, Denne M, Goldstine J, Kadish B, et al.: Polyethylene Glycol Exposure with Antihemophilic Factor (Recombinant), PEGylated (rurioctocog alfa pegol) and Other Therapies Indicated for the Pediatric Population: History and Safety.Pharmaceuticals (Basel). 2018; 11 (3). PubMed Abstract | Publisher Full Text

**Answer: **Thank you for the recommendation. We have added an additional description related to safety based on the suggested reference.
